# Parvalbumin interneuron-mediated neural disruption in an animal model of postintensive care syndrome: prevention by fluoxetine

**DOI:** 10.18632/aging.202684

**Published:** 2021-02-22

**Authors:** Yong Wang, Xiao-yu Yin, Xue He, Chen-mao Zhou, Jin-chun Shen, Jian-hua Tong

**Affiliations:** 1 Department of Anesthesiology, Pain and Perioperative Medicine, The First Affiliated Hospital of Zhengzhou University, Zhengzhou, China; 2 Department of Anesthesiology, Jinling Hospital, School of Medicine, Nanjing University, Nanjing, China; 3 Department of Anesthesiology, The Second Affiliated Hospital, Nanjing Medical University, Nanjing, China

**Keywords:** stress, behavioral outcomes, interneuron, network activity, fluoxetine

## Abstract

Postintensive care syndrome (PICS) is defined as a new or worsening impairment in cognition, mental health, and physical function after critical illness and persisting beyond hospitalization, which is associated with reduced quality of life and increased mortality. Recently, we have developed a clinically relevant animal model of PICS based on two-hit hypothesis. However, the underlying mechanism remains unclear. Accumulating evidence has demonstrated that hippocampal GABAergic interneuron dysfunction is implicated in various mood disorders induced by stress. Thus, this study investigated the role of hippocampal GABAergic interneurons and relevant neural activities in an animal model of PICS. In addition, we tested whether fluoxetine treatment early following combined stress can prevent these anatomical and behavioral pathologies. In the present study, we confirmed our previous study that this PICS model displayed reproducible anxiety- and depression like behavior and cognitive impairments, which resembles clinical features of human PICS. This behavioral state is accompanied by hippocampal neuroinflammation, reduced parvalbumin (PV) expression, and decreased theta and gamma power. Importantly, chronic fluoxetine treatment reversed most of these abnormities. In summary, our study provides additional evidence that PV interneuron-mediated hippocampal network activity disruption might play a key role in the pathology of PICS, while fluoxetine offers protection via modulation of the hippocampal PV interneuron and relevant network activities.

## INTRODUCTION

Medical advancements in the intensive care unit (ICU) have led to a substantial reduction in mortality rates in survivors of critical illness [1]. However, survivors frequently experience postintensive care syndrome (PICS), which refers to physical, cognition, and mental impairments that occur during ICU stay, after ICU discharge, as well as long-term poor prognosis beyond their ICU admission [2]. It is estimated that more than 50% of these survivors of critical illness will experience at least one symptom of PICS, which not only diminishes life quality of affected patients, but also poses a substantial issue in public health because of the increased prevalence in modern society [3, 4]. Therefore, more studies are needed to better understand the pathophysiology of PICS.

Chronic stress is one of the risk factors for the development of many neuropsychiatric diseases [5]. In patients admitted to ICU, they consistently face tremendous physical and psychological stressors, including social isolation, chemical and physical restraints, sleep disturbances due to exposure to noise and lights as well as other stressors [6, 7]. An earlier study reported that nearly 50% of patients experience symptoms of depression, anxiety, post-traumatic stress disorder, and cognitive impairment, which may persist for years [8]. Based on these findings, we have recently developed one clinically relevant animal model of PICS based on two-hit conception [9]. The “two-hit” hypothesis states that initial insult sensitizes the vulnerable brain to subsequent stress so that the two hits have synergistic toxic effects. Because bacterial infection remains the leading cause of ICU admission by epidemiological study [10], we thus subjected animals to lipopolysaccharide (LPS) injection as the first hit and subsequent chronic unpredictable stress as the second hit [9]. This model displayed reproducible anxiety- and depression like behavior and cognitive impairments, which mimics clinical features of human PICS [9]. However, the neural mechanism remains largely to be elucidated.

Accumulating evidence has documented that corticolimbic GABAergic dysfunction is implicated in the etiology of mood disorders, including schizophrenia, depression, and other related neuropsychiatric diseases [11–14]. Thus, the present study investigated whether GABAergic interneurons and its relevant neural activities are affected by our recently established animal model of PICS [9]. In addition, we examined whether pharmacological intervention with serotonergic system by fluoxetine, a selective serotonin reuptake inhibitor [15], could attenuate the neural and behavioral disturbances of PICS. In particular, we focused our interest on the hippocampus because this brain region plays a critical role in stress, emotion, and affective behaviors, and is susceptible to chronic stress exposure [5].

## RESULTS

### Survival rate

**Figure 1:**
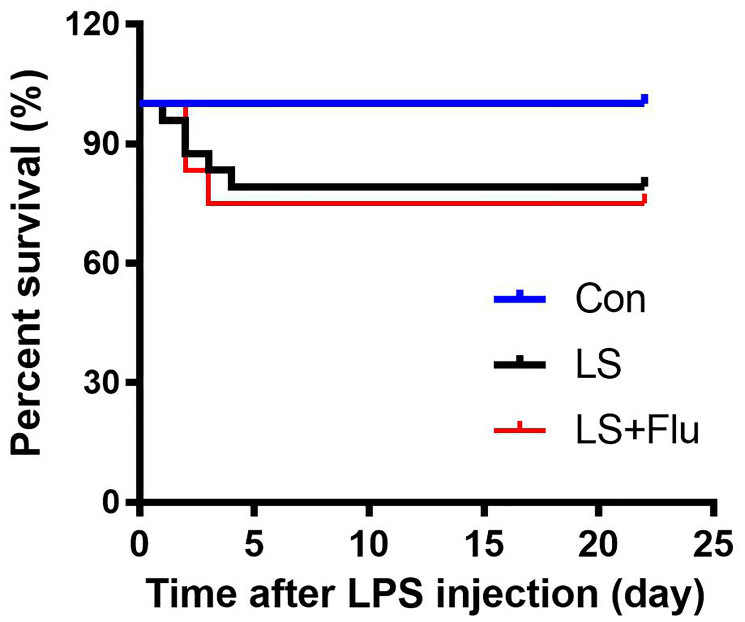
**Survival rate.** No animals died in the control group, the survival rate was 79.167% in LS group and 75% in LS + fluoxetine group (*n* = 18-24). Con, control; lipopolysaccharide; LPS, Flu, fluoxetine; LS, LPS + stress.

To observe the effects of combined stress on mortality rate, we recorded survival rate during the experimental period. As shown in Figure 1, no animals died in the control group (100% for control group). Animal death was observed in the first 4 days following combined stress protocol, but there was no difference in survival rate among groups (*P* = 0.089). The survival rate was 79.167% in LPS + stress (LS) group and 75% in LS + fluoxetine group.

### Hippocampal inflammatory response induced by combined stress was attenuated by fluoxetine treatment

**Figure 2:**
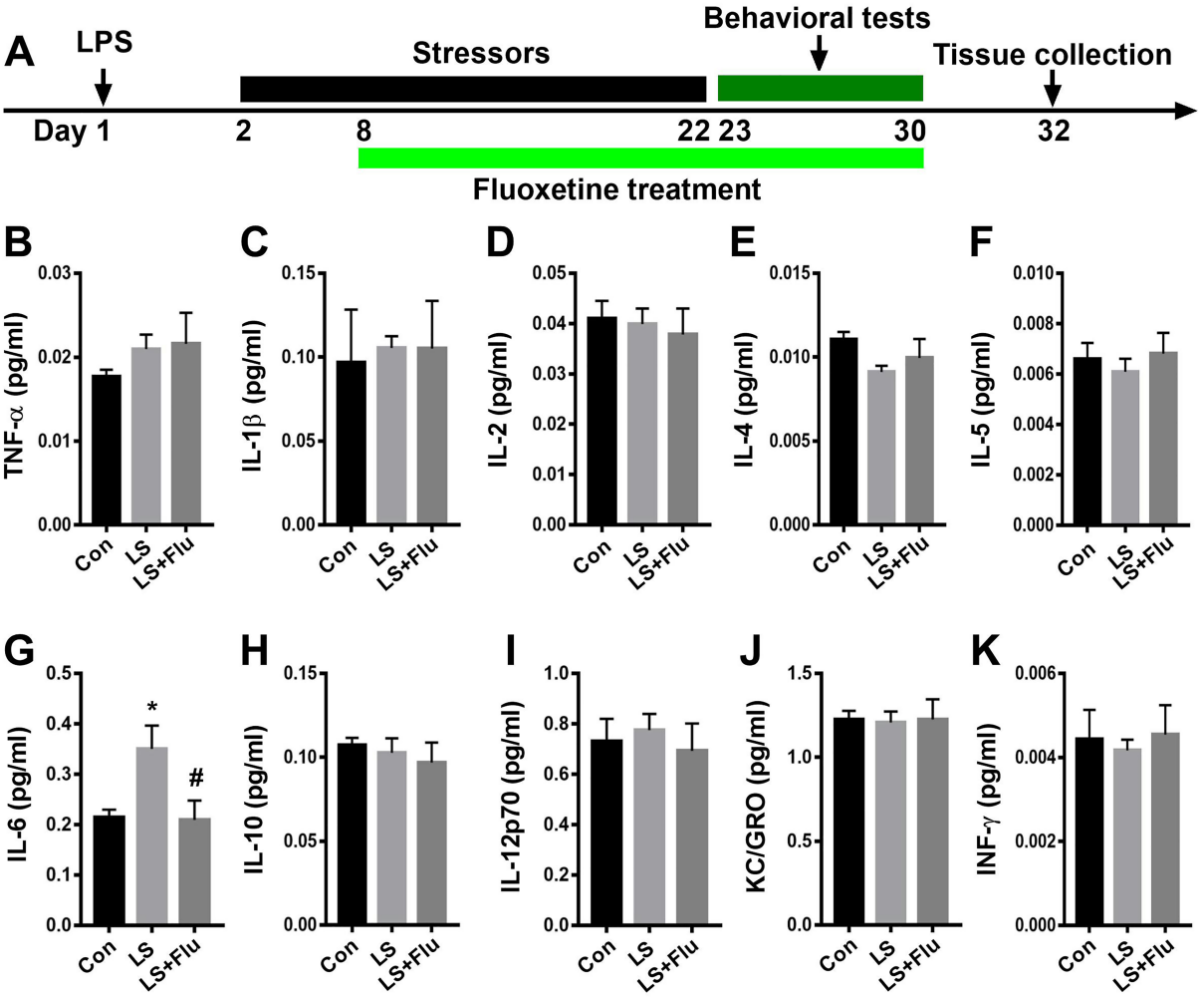
**Fluoxetine treatment attenuated IL-6 level after combined stress.** (**A**) Schematic timeline of the experimental procedure. (**B–K**) Quantification of inflammatory mediators in the hippocampus. Data are shown as mean ± SEM (*n* = 6), ^*^P < 0.05 vs control group, ^#^*P* < 0.05 vs LS group. Con, control; LPS, lipopolysaccharide; Flu, fluoxetine; LS, LPS + stress.

To determine changes in immune response in the hippocampus after combined stress, we performed immunostaining by using antibodies of IBA1 or GFAP: as well as MSD for inflammatory mediator measurements. The schematic timeline of the experimental procedure was shown in Figure 2A. Relative to control group, the intensity of IBA1 cells in the hippocampus increased significantly in LS group. Similarly, the intensity of hippocampal GFAP cells was significantly increased in LS group compared with control group. For inflammatory mediator measurements, we observed a significantly increased IL-6 level in the hippocampus in LS group than that in control group. These results suggested that combined stress induced an enhanced inflammatory reaction. However, chronic treatment with fluoxetine only reversed the intensity of IBA1 (CA1: F_(2, 9)_ = 9.236, *P* = 0.0066; CA3: F_(2, 9)_ = 5.029, *P* = 0.0342; DG: F_(2, 9)_ = 11.56, *P* = 0.0033, Supplementary Figure 1) and GFAP (CA1: F_(2, 9)_ = 22.35, *P* = 0.0003; CA3: F_(2, 9)_ = 5.993, *P* = 0.0221; DG: F_(2, 9)_ = 0.6033, *P* = 0.5677, Supplementary Figure 2) of the CA1 region. The increased hippocampal IL-6 level in the LS group was also prevented by fluoxetine treatment (F_(2, 15)_ = 4.964, *P* = 0.0222, Figure 2G). There was no difference in TNF-α (F_(2, 15)_ = 0.7828, *P* = 0.4749, Figure 2B), IL-1β (F_(2, 15)_ = 0.04009, *P* = 0.9608, Figure 2C), IL-2 (F_(2, 15)_ = 0.1656, *P* = 0.8489, Figure 2D), IL-4 (F_(2, 15)_ = 1.738, *P* = 0.2094, Figure 2E), IL-5 (F_(2, 15)_ = 0.3083, *P* = 0.7393, Figure 2F), IL-10 (F_(2, 15)_ = 0.341, *P* = 0.7164, Figure 2H), IL-12p70 (F_(2, 15)_ = 0.2097, *P* = 0.8132, Figure 2I), KC/GRO (F_(2, 15)_ = 0.01621, *P* = 0.9839, Figure 2J), or INF-γ (F_(2, 15)_ = 0.1064, *P* = 0.8997, Figure 2K) in the hippocampus among groups.

### PV interneuron deficit induced by combined stress was attenuated by fluoxetine treatment

**Figure 3:**
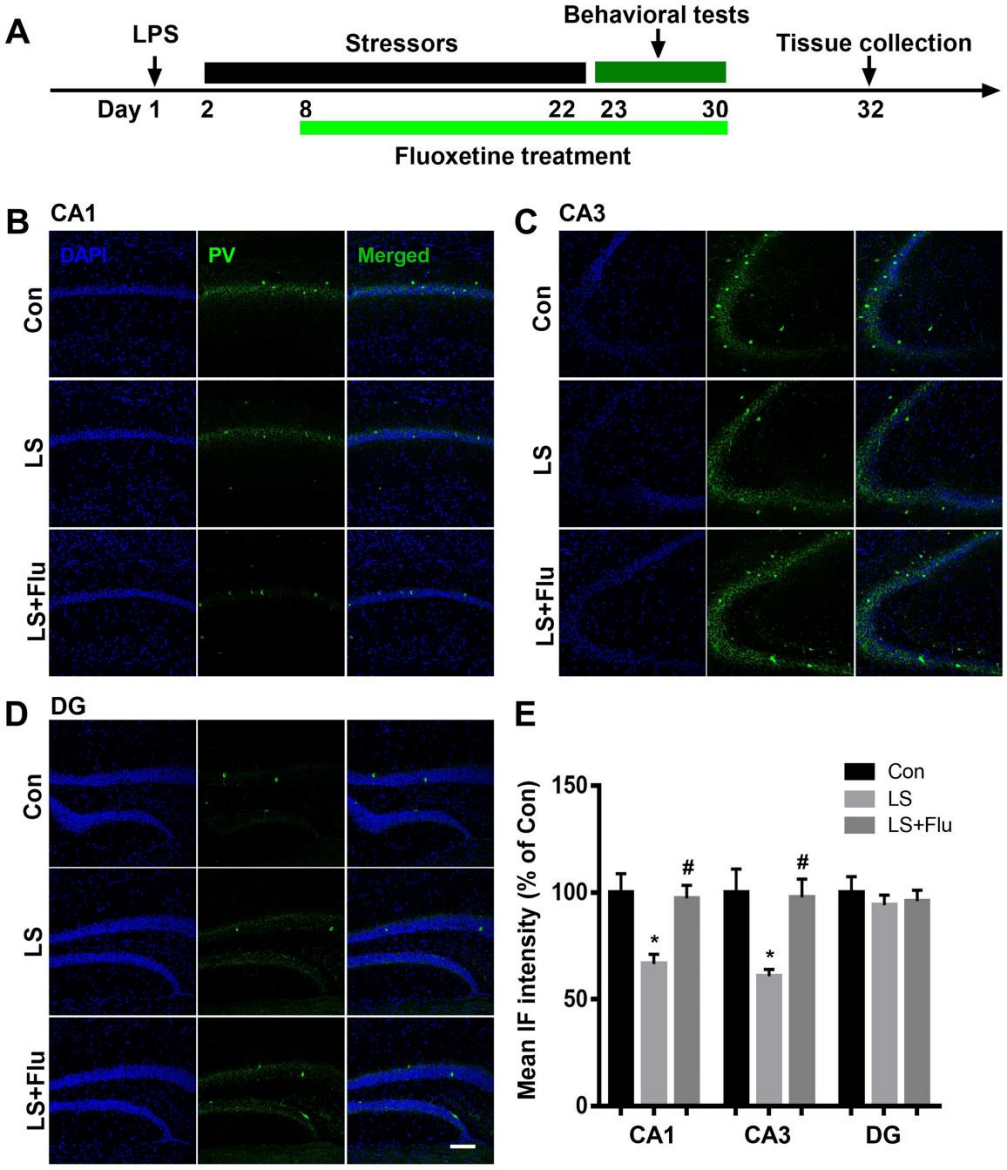
**Fluoxetine treatment attenuated PV deficit after combined stress.** (**A**) Schematic timeline of the experimental procedure. (**B–D**) Representative images of PV interneurons in all subregions of the hippocampus. (**E**) Quantification of mean PV immunofluorescence in the hippocampus. Data are shown as mean ± SEM (*n* = 4), ^*^P < 0.05 vs control group, ^#^*P* < 0.05 vs LS group, scale ba*r* = 100 μm. Con, control; LPS, lipopolysaccharide; Flu, fluoxetine; IF, immunofluorescence; LS, LPS + stress.

**Figure 4:**
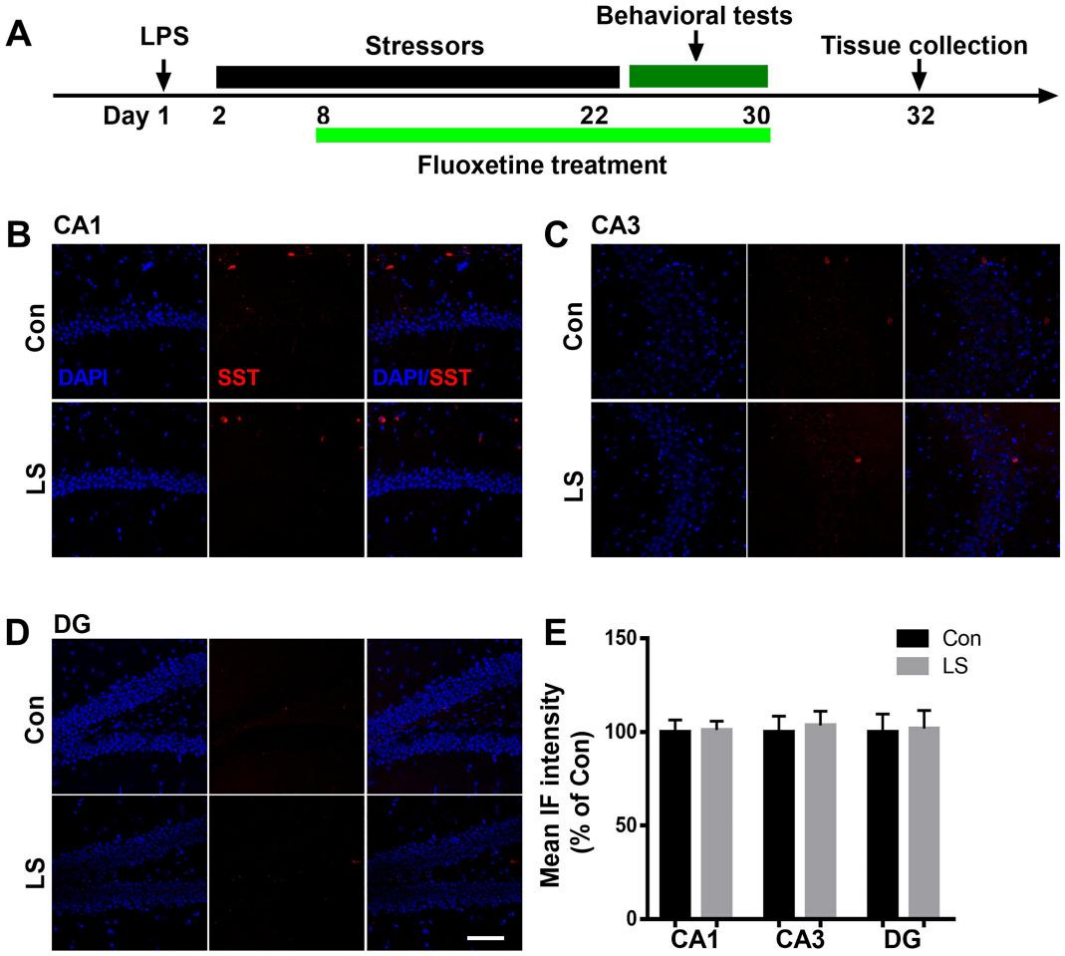
**Combined stress did not affect SST level.** (**A**) Schematic timeline of the experimental procedure. (**B–D**) Representative images of SST interneurons in all subregions of the hippocampus. (**E**) Quantification of mean SST immunofluorescence in the hippocampus. Data are shown as mean ± SEM (*n* = 4), scale ba*r* = 100 μm. Con, control; LPS, lipopolysaccharide; Flu, fluoxetine; IF, immunofluorescence; LS, LPS + stress.

To evaluate GABAergic interneuron changes in the hippocampus after combined stress, we performed immunostaining by antibodies raised against PV or SST, two major subgroups of GABAergic interneurons. As shown in Figure 3, the intensity of PV was significantly decreased in the CA1 and CA3 regions of the hippocampus in LS group compared with control group, which were reversed by fluoxetine treatment (CA1: F_(2, 9)_ = 7.65, *P* = 0.0115; CA3: F_(2, 9)_ = 7.252, *P* = 0.0133). There was no difference in PV intensity of DG among groups (F_(2, 9)_ = 0.271, *P* = 0.7686). Surprisingly, we found combined stress did not affect SST (CA1: *t* = 0.1253, *P* = 0.9044; CA3: *t* = 0.3095, *P* = 0.7674; DG: *t* = 0.1282, *P* = 0.9022, Figure 4) expression in all regions of the hippocampus. These results suggested this combined stress protocol selectively impaired hippocampal PV interneurons.

### Altered hippocampal neural oscillations induced by combined stress were reversed by fluoxetine treatment

**Figure 5:**
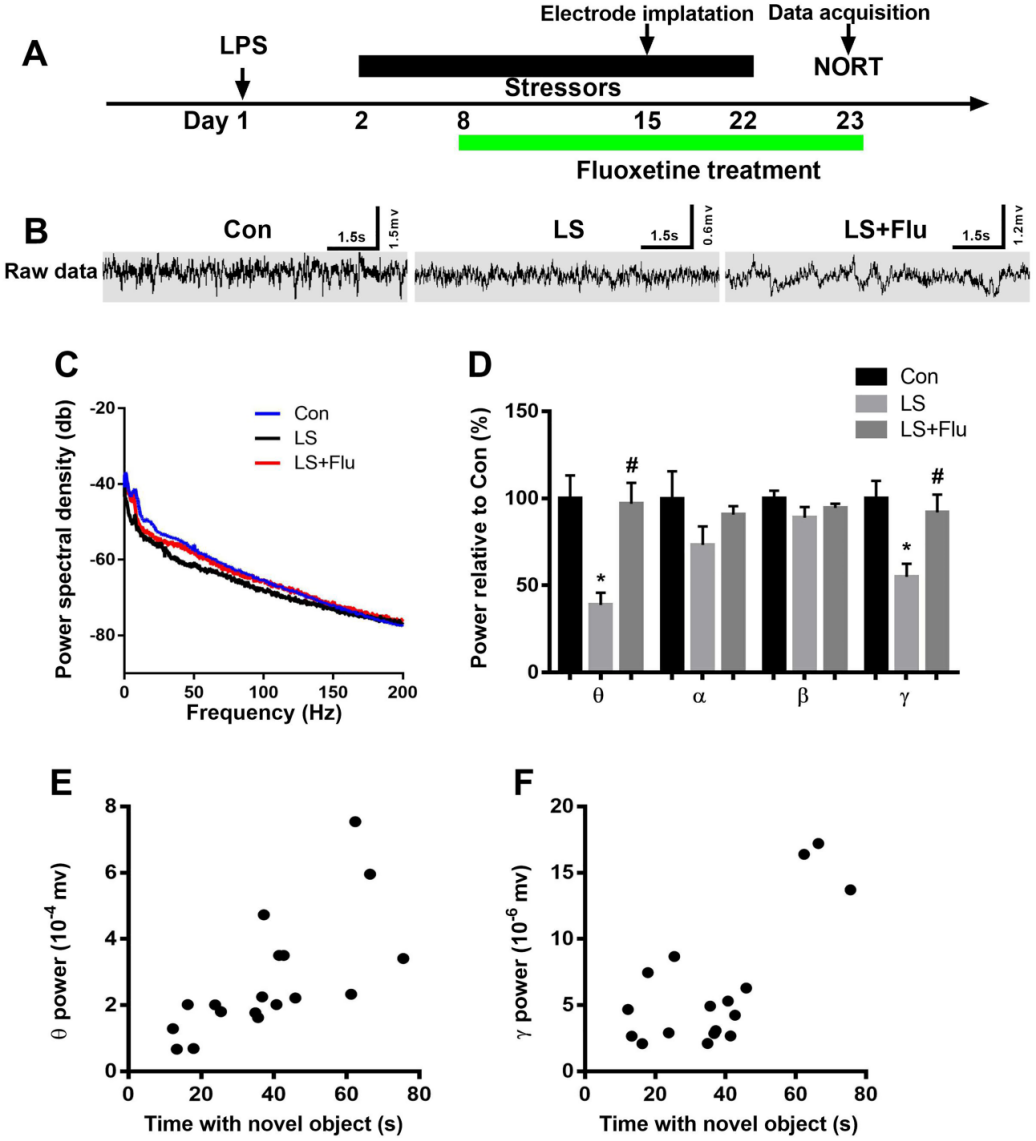
**Altered hippocampal neural oscillations induced by combined stress were reversed by fluoxetine treatment.** (**A**) Schematic timeline of the experimental procedure. (**B**) Representative images of local field potential in the CA1 of the hippocampus. (**C**) Quantification of local field potential in the CA1 of the hippocampus. (**D**) Quantification of average theta, alpha, beta, and gamma power in the CA1 of the hippocampus. (**E**) Theta oscillation was positively correlated with time spent with novel object. (**F**) Gamma oscillation was positively correlated with time spent with novel object. Data are shown as mean ± SEM (n = 6), ^*^P < 0.05 vs control group, ^#^*P* < 0.05 vs LS group. Con, control; LPS, lipopolysaccharide; Flu, fluoxetine; LS, LPS + stress; NORT, novel object recognition test.

To further evaluate the causal role of altered oscillatory activities in the symptoms of PICS, we recorded LFP during novel object exploration test. The schematic timeline of the experimental procedure was shown in Figure 5A. Figure 5B showed representative images of local field potential and Figure 5C displayed the power spectral density in the CA1 of the hippocampus. Power spectral analysis showed that combined stress induced significantly decreased theta and gamma power when compared with control group. However, fluoxetine treatment reversed these deficits (theta: F_(2,15)_ = 9.693, *P* = 0.002; alpha: F_(2,15)_ = 1.415, *P* = 0.2735; beta: _(2,15)_ = 1.45, *P* = 0.2656; gamma power: F_(2,15)_ = 6.607, *P* = 0.0088, Figure 5D). In addition, linear regression analysis showed that theta or gamma oscillation was positively correlated with time spent with novel object (theta: *r* = 0.6921, *P* = 0.0015; gamma: *r* = 0.7242, *P* = 0.001, Figure 5E–5F). These data suggested that deficits in theta and gamma play an important role in cognition impairment.

### Abnormal behavioral outcomes induced by combined stress were reversed by fluoxetine treatment

**Figure 6:**
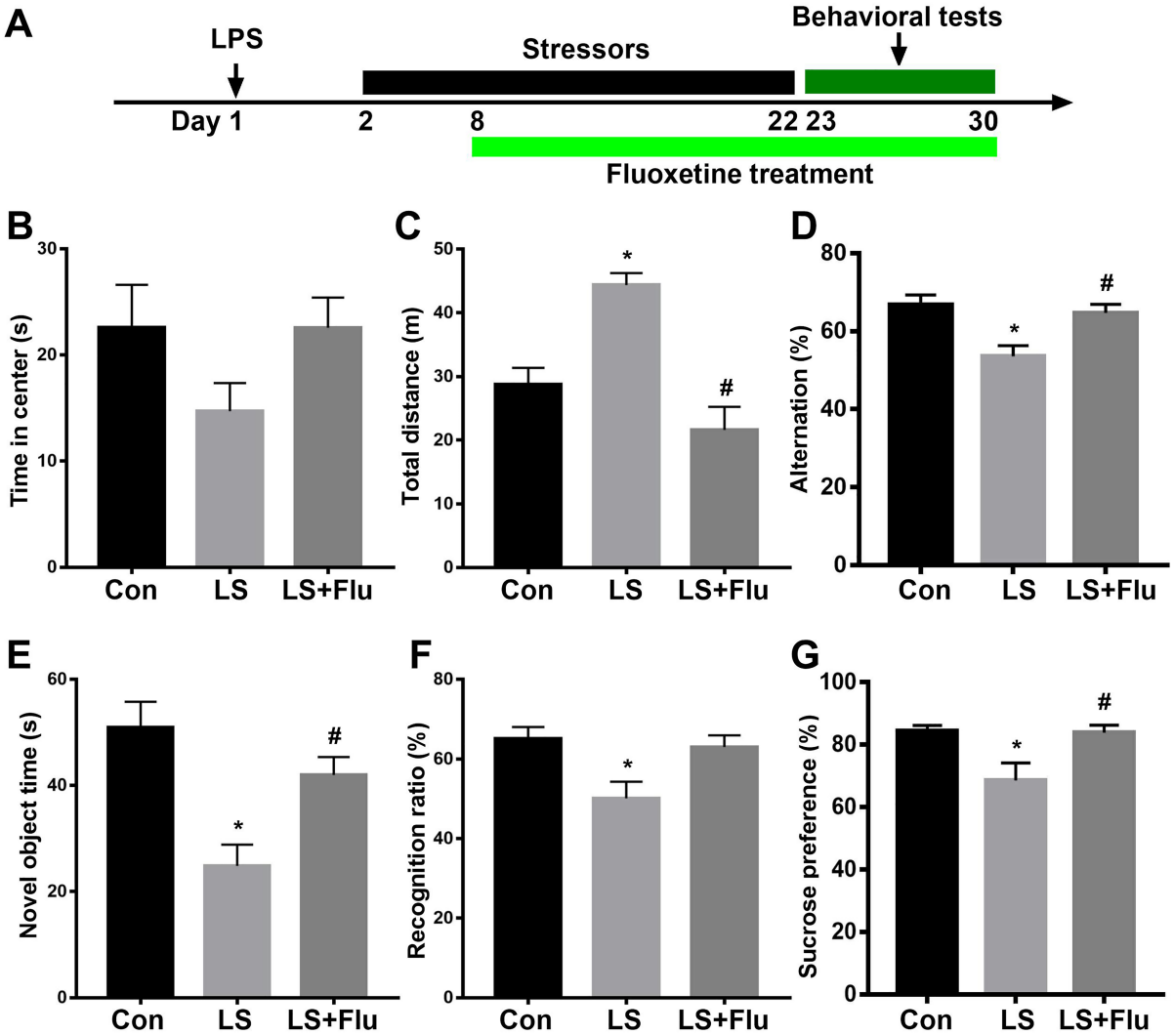
**Abnormal behavioral outcomes induced by combined stress were reversed by fluoxetine treatment.** (**A**) Schematic timeline of the experimental procedure. (**B**) There was no difference in time spent in the center in the open arena among groups. (**C**) Combined stress significantly increased distance travelled compared with control group, which was prevented by fluoxetine treatment. (**D**) Mice in LS group displayed significantly decreased spontaneous alteration than that in control group, which was reversed by fluoxetine treatment. (**E**) Combined stress induced significantly decreased time with the novel object, which was prevented by fluoxetine treatment. (**F**) Combined stress induced significantly decreased recognition ratio was not prevented by fluoxetine treatment. (**G**) Combined stress induced significantly decreased preference for sucrose was prevented by fluoxetine treatment. Data are shown as mean ± SEM (n = 12), ^*^P < 0.05 vs control group, ^#^*P* < 0.05 vs LS group. Con, control; LPS, lipopolysaccharide; Flu, fluoxetine; LS, LPS + stress.

The schematic timeline of the experimental procedure was shown in Figure 6A. The open field test was performed to investigate locomotor activity and anxiety-like behavior. Although treatment reversed these deficits (theta: F_(2,15)_ = 9.693, *P* = 0.002; alpha: F_(2,15)_ = 1.415, *P* = 0.2735; beta: _(2,15)_ = 1.45, *P* = 0.2656; gamma power: F_(2,15)_ = 6.607, *P* = 0.0088, Figure 5D). In addition, linear regression analysis showed that theta or gamma oscillation was positively correlated with time spent with novel object (theta: *r* = 0.6921, *P* = 0.0015; gamma: *r* = 0.7242, *P* = 0.001, Figure 5E–5F). These data suggested that deficits in theta and gamma play an important role in cognition impairment. combined stress did not affect time spent in the center of the open arena (F_(2, 33)_ = 1.988, *P* = 0.1531, Figure 6B), it significantly increased the distance travelled as compared with the control group. This increase was prevented by fluoxetine treatment (F_(2, 33)_ = 17.32, *P* < 0.0001, Figure 6C). Next, mice were tested in the spontaneous alternation Y-maze paradigm that assesses spatial working memory. The mice in LS group displayed significantly decreased spontaneous alteration than that in control group, which was reversed by fluoxetine treatment (F_(2, 33)_ = 8.044, *P* = 0.0014, Figure 6D). In the novel object recognition test, combined stress significantly decreased their exploration time with novel object (F_(2, 33)_ = 10.35, *P* = 0.0003, Figure 6E) and novel object recognition ratio (F_(2, 33)_ = 5.674, *P* = 0.0076, Figure 6F) compared with control group, while the decreased exploration time with novel object in LS group was prevented by fluoxetine treatment. In the sucrose preference test, mice showed significantly decreased preference for sucrose than controls, which was prevented by fluoxetine treatment (F_(2, 33)_ = 5.94, *P* = 0.0063, Figure 6G).

## DISCUSSION

By using this clinically relevant animal model of PICS, we confirmed our previous finding that this PICS model displayed reproducible anxiety- and depression like behavior and cognitive impairments. More importantly, we found disturbed hippocampal PV interneuron and neural network in PICS, which can be precluded by fluoxetine treatment. Thus, our study reveals a mechanism of disturbed inhibitory neural network that may contribute to abnormal behavioral responses of PICS and also the efficacy of fluoxetine treatment. Although the mortality of ICU patients has declined significantly in recent decades, morbidity after ICU discharge remains a significant concern [16]. It is important to point out that PICS is being increasingly recognized as a complication that affects more than 50% of critically ill patients, which has a profound impact on patients’ lives, including reduced quality of life and increased mortality [1–3]. With improvements in healthcare and increased numbers of ICU survivors, PICS is likely to become more prevalent and will continue to be a public health issue [6]. Although PICS is a multidimensional concept, each component has been studied separately. It is estimated that more than 50% of all ICU survivors suffer from at least one or more PICS-related impairment [2]. Among them, depression, anxiety, cognitive impairment, and posttraumatic stress disorder related symptoms are the main components of psychological PICS [2, 3, 8, 17, 18]. Using a translational mouse model of PICS, we have recently shown that combined immune challenge with LPS and subsequent chronic stress induces synergistic pathological effects on behavioral outcomes [9], which were consistent with one recent study in which they showed depressive-like behavior in a two-hit model of depression by using LPS injection and subsequent chronic unpredictable mild stress protocol [19]. Although cognition domain such as attention is not investigated in the present study, we confirmed our previous finding that this two-hit hypothesis model of PICS displayed reproducible anxiety- and depression like behavior and cognitive impairments, suggesting this model is reliable. However, the underlying neural mechanism remains fully to be elucidated.

Environmental factors, such as trauma and stressful life events, profoundly alter neural structure and functional plasticity of the brain, drives changes in physiology and behavior, and contributes to a variety of mental disorders [5]. Although the mechanism underlying PICS remains largely unexplored, the pathobiology of psychiatric conditions is closely linked to inflammatory processes [20–22]. Similarly, we showed signs of hippocampal inflammation in the form of glia overactivation (increased Iba1 and GFAP expressions) and hypersecretion of inflammatory cytokines as reflected by MSD measurement. Thus, our study provides additional evidence that neuroinflammation may be a common mechanism contributing to stress-induced mental disorders. Of note, fluoxetine treatment down-regulated neuroinflmmation and reversed some of the neurobehavioral abnormalities, suggesting neuroinflammation might play an initial and decisive role in PICS. Indeed, earlier studies have shown that chronic stress is associated with a chronic, low-grade inflammation in other models of neurodegenerative diseases [20–23]. Since we used a combined stress protocol, LPS challenge may act as an early toxic event and render the brain susceptible to subsequent chronic stress. Indeed, the synergistic relationship between inflammation and stress has been well described, in which stress can prime the immune system and thereby elicit an exaggerated response to a subsequent inflammatory stimulus and vice versa [23]. One question that remains unanswered is the underlying mechanism by which neuroinflammation induced symptoms of PICS.

Increasing evidence has suggested that cortical GABAergic interneurons play crucial roles in diverse brain functions [24]. The most extensively studied inhibitory interneurons are those that express PV or SST, which comprise the majority of GABAergic system and exhibit distinct molecular and physiological properties [25]. There is extensive evidence that stress disrupts the structural integrity of specific GABAergic interneurons and this also affects the functioning of hippocampal GABAergic networks [26–28]. Preclinical data has shown that hippocampal PV or SST interneurons are vulnerable to psychosocial stressors, thus PV and SST deficits are frequently observed pathological features in depression and other neurological disorders with mood disturbances [29–32]. In our study, we evaluated these two subpopulations of GABAergic interneurons. Surprisingly, we found a reduction in the hippocampal PV but not SST interneurons in our model. Our data was in disagreement with previous findings, in which they showed that dysfunction of SST interneurons is likely associated with the pathophysiology of many mental disorders [33–35]. This discrepancy is likely to be due to differences in the two stress paradigms used, i.e. our study protocol includes initial immune challenge and is different from those induced by chronic stress. Overall, we provide further evidence supporting the role of GABAergic interneurons, especially hippocampal PV interneurons in the pathophysiology of PICS.

More importantly, these diverse subtypes of GABAergic interneurons play a key role in cognitive process as they provide networks of inhibition and orchestrate network oscillations [24, 36]. In particular, PV interneurons are implicated in mediating synchronization of oscillatory activities [24, 37]. By contrast, abnormal brain rhythms are considered to be as potential pathophysiological mechanisms causing mental diseases [38]. In further support of a link between PV interneurons and symptoms of PICS, we found that theta or gamma oscillation, a neurophysiological phenomenon that is supported by PV interneuron function, correlates positively with time with novel object. In one recent study, it is reported that PV and SST interneurons play a key role in hippocampal theta-nested gamma oscillations and long-term potentiation [39]. This is further supported by findings that changes in cortical gamma oscillation power and/or frequency can lead to various behavioral and cognitive effects [24, 37]. However, our study did not find a relation between these oscillations and anxiety or depression-like behavior. One explanation is likely to be that neuronal oscillation in the theta or gamma frequency range in the dorsal hippocampus is important for cognition performance but not for anxiety or depression-like behavior. Thus, our study provides a correlative link between behavioral alterations in PICS mice and the possible underlying cellular and network mechanisms.

Since GABAergic system has been proposed as a potential therapeutic target of antidepressant, we examined whether treatment with the antidepressant fluoxetine offered protection by attenuating the neural and behavioral disturbances. Several studies have demonstrated that treatment with fluoxetine reverses the functional and structural impairments of the hippocampal formation induced by stress [9, 15, 40]. Although fluoxetine is designed to normalize monoaminergic transmission, it is now become widely accepted that its antidepressant mechanisms do not simply by increasing monoamine transmitter function and that alterations in these transmitter systems are not sufficient to explain the complex nature of affective disorders. There is accumulating evidence suggesting chronic fluoxetine treatment can increase GABAergic tone in the brain [41–43]. This is further confirmed by one recent study demonstrating that 5-HT_5A_ signaling in PV interneurons mediates delayed antidepressant action of fluoxetine [44]. In addition, it has been demonstrated that antidepressant like fluoxetine mediated alteration of oscillations in the CA1 area and resulted in consequent cognitive effects [45]. In the current study, we showed that chronic fluoxetine treatment restored hippocampal PV interneuron deficit and alteration of oscillations, contributing to improved neurobehavioral outcomes. Our data together with previous findings suggested that chronic fluoxetine treatment offer protection via modulation of hippocampal PV interneurons and relevant network activities. However, the effect of the fluoxetine should be also investigated under physiological conditions in our future study.

## CONCLUSIONS

In summary, our study suggested that PV interneuron-mediated hippocampal network activity disruption might play a key role in the symptoms of PICS, while fluoxetine offers neuroprotection by reversing these abnormities. Thus, our data provide a mechanistic link between combined stress, hippocampal neuroinflammation, PV interneuron deficit, neural network disturbance, and symptoms of PICS. However, more specific studies are needed to confirm our results.

## MATERIALS AND METHODS

### Animals

Seventy male C57BL/6 mice (3–4 months) were obtained and purchased from the animal center of Jinling Hospital, Nanjing University, Nanjing, China. Mice were housed under standard conditions in groups of three to four per cage in a temperature 24 ± 2°C, humidity 55 ± 10% with access to water and food. Experimental protocols and monitoring for suffering were treated according to the National Institute of Health Guidelines on the use of laboratory animal and with approval of the Animal Care and Use Committee of The First Affiliated Hospital of Zhengzhou University, Zhengzhou, China (ZD20190825).

### Combined stress protocol

The combined stress protocol was selected as we described previously [9]. It is based on a two-hit conception, in which LPS injection as the first hit and subsequent modified chronic unpredictable stress as the second hit. Briefly, LPS (serotype 0111: B4, Sigma, Lot # 064M4125V, Shanghai, China) was diluted in 0.9% sterile saline and given intraperitoneally (*i.p.,* 3 mg/kg) in a final injection volume of 0.2 ml. This dose of LPS induces moderate sepsis severity, which is reflected by ~20% mortality rate following LPS injection. In the control group, mice received the same volume of 0.9% sterile saline to control for stress effect. For stress exposure, we selected certain relevant stressors that frequently exist in critically ill patients living in the ICU. As our previous study described [9], animals were housed singly and exposed to four of the following stressors daily in a random order for 21 consecutive days (Supplementary Table 1). The control, non-stressed mice groups were reared under similar conditions but received no stressors. Because our previous study showed that LPS only group did not result in neurobehavioral abnormities [9], we did not include this group in the present study.

### Drug treatment

To mimic a realistic situation of antidepressant intervention, we administered fluoxetine (Tocris Bioscience, Bristol, UK) for a clinically relevant period of 4 weeks. Thus, fluoxetine was dissolved in drinking water and given 1 week after LPS injection until the end of the behavioral tests. The solutions were prepared according to the mouse average weight and daily water consumption in order to provide an average daily intake of 20 mg/kg.

### Behavioral experiments

Mice were transported and left for habituation to the testing room for 1 h prior to the behavioral tests as we previously described [9]. The order of the behavioral tests was open field, Y maze, novel object recognition, and sucrose preference tests. All behavioral tests were carried out between 9 and 12 AM on the designated day of experiment.

### Open field test

The open field apparatus is made of a 50 × 50-cm rectangular arena and 40-cm-high walls. Each mouse was placed in the centre of the arena and was tested for general exploratory locomotion for 5 min. The total distance traveled in the arena and the time spent in central zones was scored using a computerized video-tracking system and software (XR–XZ301, Shanghai Softmaze Information Technology Co., Ltd., Shanghai, China). The apparatus was cleaned by using 70% ethanol after each mouse was tested.

### Y-maze

The Y-maze was used to evaluate spatial working memory [46], which was performed in a black Plexiglas Y-Maze with three arms (30 cm long × 14 cm wide × 15 cm high) at 120° angles, designated A, B, and C. Each mouse was placed in the center of Y-Maze facing arm A and allowed to explore all three arms of the maze freely for 8 min. The sequence and total number of arms entered were recorded. Alternations were calculated when a mouse consecutively traveled to the three arms without re-entering the previously visited arms. The alternation rate was calculated using the following formula: Alternation rate (%) = number of alternations/(total number of arms entries - 2) × 100%.

### Novel object recognition test

The novel object recognition (NOR) task was performed in an open field (40 cm × 60 cm wide × 50 cm tall) with three objects, two of which were almost the same, the other was different. Mice were habituated in NOR arena for 10 min in absence of testing objects for two days. Twenty-four hours later, the animal was exposed to two familiar objects for 10 min. To avoid a preference for one side of the open field, two familiar objects were counterbalanced between each mouse. In the testing trial, one of the objects was changed into a novel object with different color and shape. The exploration of the new object and familiar object was recorded by a video-tracking system for 10 min. The discrimination score for novel object exploration ratio was calculated with the following formula: time exploring novel object/(time exploring novel object + time exploring familiar object) × 100%. Equipment and apparatus were cleaned using 70% ethanol between trials.

### Sucrose preference test

Sucrose preference test is a well-accepted behavioral test measuring an anhedonia-like state. Anhedonia was measured by preference for a sucrose solution over water, using a two-bottle free choice method. Briefly, each mouse was presented simultaneously with two bottles, one with 1% sucrose solution and the other containing tap water. Mice were then given a free choice between either tap water or 1% sucrose in tap water solution for 24 h. After 12 h, the position of the two bottles was switched to control for a side preference in drinking behavior. Twenty-four hours later, the bottles were then weighed to measure how much liquid was consumed. The sum of water and sucrose intake was defined as the total intake, and sucrose preference was expressed as the percentage of sucrose intake from total intake. Sucrose preference was calculated as sucrose consumption/(sucrose consumption + water consumption) × 100%).

### Immunofluorescence

Mice were deeply anesthetized with 2% sodium pentobarbital in saline (60 mg/kg, *i.p.*) and transcardially perfused with phosphate-buffered saline (PBS, pH 7.4), followed by 4% iced phosphate-buffered paraformaldehyde (PFA). Brains were removed and postfixed in the 4% PFA for 12 h and dehydrated in 30% sucrose at 4°C overnight. Then brains were embedded in O.C.T. compound, and 30-μm-thick coronal sections were obtained using a Leica cryostat (CM 3050S) and restored in –70°C for further use. Slices were initially blocked with 1–2% bovine serum albumin and 0.03% Triton X-100 for 2 h at room temperature and then incubated with the primary antibodies: rabbit anti-ionized calcium binding adapter molecule 1 (IBA1, 1:1000; WAKO, 019-19741), rabbit anti-glial fibrillary acidic protein (GFAP: 1:200, Proteintech, 16825-1-AP), rabbit anti-PV (1:500; Abcam, ab11427), or rat anti-SST (1:500; Abcam, ab30788) overnight at 4°C. Following washing 3 × 5 min in 1 × PBS, sections were incubated with secondary antibodies for 2 h at room temperature. After washing out the secondary antibodies (Cy3-conjugated donkey anti-rat IgG (1:300; Santa Cruz Biotechnology, Dallas, TX) in 1 × PBS, sections were mounted on slides with 4′, 6-diamidino-2-pheny-lindole (DAPI) for 10 min. Detailed images were taken on an Olympus FV1000 confocal microscope. The intensities were calculated by Image J software (National Institutes of Health, Bethesda, MD, USA).

### Meso scale discovery (MSD)

Inflammatory cytokines in the hippocampus were determined using multiplex biomarker assay platform from MSD, according to manufacturer's instructions. Diluted protein extracts (50 ul) were loaded into a 96 well plates, along with standards. Plates were then sealed and incubated at room temperature for 2 h followed by three washes with phosphate buffered saline Tween. After adding the detection antibodies, plates were sealed and incubated for a further 2 h. Then read solution was added following three washes with PBST, and plates were immediately read using an MSD plate reader. The concentrations of inflammatory cytokines were expressed by pg/ml.

### *In vivo* electrophysiology

For local field potential (LFP) recording, mice underwent an implant surgery as we previously described [47]. Briefly, mice were anesthetized with phenobarbital sodium (*i.p.,* 40 mg/kg) and placed in a stereotaxic frame with precision micromanipulators. After craniotomy and removal of dura, an 8-channel linear silicon probes were used to record right CA1 region of the hippocampus. The coordinates were determined according to the mouse brain atlas in stereotaxic coordinates (posterior, 2.1 mm; lateral, 1.5–1.7 mm; depth, 1.7–2.1 mm). LFPs were recorded while the mice underwent the novel object recognition test. The signals were filtered with a pass-band of 0.3–300 Hz and were further amplified and digitized at 2 kHz. The recorded LFPs were filtered by a 50 Hz notching filter to remove the powerline artifact. For LFP analysis, the wideband recordings were down-sampled at 1000 Hz. All data analyses were performed by Neuroexplorer (Plexon Inc., Dallas, TX) software.

### Statistical analysis

Data were analyzed and plotted by GraphPad Prism 7.0 (GraphPad Software, La Jolla, CA, USA). Data are presented as mean ± standard error of the mean (S.E.M.). The data were screened for normality and homogeneity of variance. For comparison of two groups, unpaired *t*-tests were used if the data were normally distributed or Mann–Whitney tests if it was not. Multiple comparisons were analyzed by one-way ANOVA followed by Tukey’s test. The survival rate was estimated by Kaplan–Meier method and compared by the log-rank test. Bivariate relationships were evaluated using Pearson correlation coefficients. A *P* < 0.05 was considered statistically significant.

### Ethics approval and consent to participate

Experimental protocols and monitoring for suffering were treated according to the National Institute of Health Guidelines on the use of laboratory animal and with approval of the Animal Care and Use Committee of The First Affiliated Hospital of Zhengzhou University, Zhengzhou, China (ZD20190825).

### Availability of data and materials

All datasets generated and/or analyzed in this study are available from the corresponding author on reasonable request.

## Supplementary Material

Supplementary Figures

Supplementary Table 1

## References

[1] Kondo Y, Fuke R, Hifumi T, Hatakeyama J, Takei T, Yamakawa K, Inoue S, Nishida O. Early rehabilitation for the prevention of postintensive care syndrome in critically ill patients: a study protocol for a systematic review and meta-analysis. BMJ Open. 2017; 7:e013828. 10.1136/bmjopen-2016-013828 28249850 PMC5353352

[2] Marra A, Pandharipande PP, Girard TD, Patel MB, Hughes CG, Jackson JC, Thompson JL, Chandrasekhar R, Ely EW, Brummel NE. Co-Occurrence of Post-Intensive Care Syndrome Problems Among 406 Survivors of Critical Illness. Crit Care Med. 2018; 46:1393–401. 10.1097/ccm.0000000000003218 29787415 PMC6095801

[3] Jackson JC, Pandharipande PP, Girard TD, Brummel NE, Thompson JL, Hughes CG, Pun BT, Vasilevskis EE, Morandi A, Shintani AK, Hopkins RO, Bernard GR, Dittus RS, et al. Depression, post-traumatic stress disorder, and functional disability in survivors of critical illness in the BRAIN-ICU study: a longitudinal cohort study. Lancet Respir Med. 2014; 2:369–79. 10.1016/s2213-2600(14)70051-7 24815803 PMC4107313

[4] Davidson JE, Harvey MA, Bemis-Dougherty A, Smith JM, Hopkins RO. Implementation of the Pain, Agitation, and Delirium Clinical Practice Guidelines and promoting patient mobility to prevent post-intensive care syndrome. Crit Care Med. 2013; 41:S136–45. 10.1097/ccm.0b013e3182a24105 23989091

[5] Notaras M, van den Buuse M. Neurobiology of BDNF in fear memory, sensitivity to stress, and stress-related disorders. Mol Psychiatry. 2020; 25:2251–2274. 10.1038/s41380-019-0639-2 31900428

[6] Luetz A, Grunow JJ, Mörgeli R, Rosenthal M, Weber-Carstens S, Weiss B, Spies C. Innovative ICU Solutions to Prevent and Reduce Delirium and Post-Intensive Care Unit Syndrome. Semin Respir Crit Care Med. 2019; 40:673–86. 10.1055/s-0039-1698404 31826268

[7] Lee M, Kang J, Jeong YJ. Risk factors for post-intensive care syndrome: A systematic review and meta-analysis. Aust Crit Care. 2020; 33:287–294. 10.1016/j.aucc.2019.10.004 31839375

[8] Harvey MA. The truth about consequences--post-intensive care syndrome in intensive care unit survivors and their families. Crit Care Med. 2012; 40:2506–7. 10.1097/ccm.0b013e318258e943 22809925

[9] Mao M, Li S, Zong M, Qiu L, Yang J, Xia J, Yang J, Ji M. Two-hit model of postintensive care syndrome induced by lipopolysaccharide challenge and subsequent chronic unpredictable stress in mice. Int Immunopharmacol. 2019; 70:446–58. 10.1016/j.intimp.2019.03.012 30856395

[10] Kahn JM, Davis BS, Yabes JG, Chang CCH, Chong DH, Hershey TB, Martsolf GR, Angus DC. Association Between State-Mandated Protocolized Sepsis Care and In-hospital Mortality Among Adults With Sepsis. JAMA. 2019; 322:240–50. 10.1001/jama.2019.9021 31310298 PMC6635905

[11] Perez SM, Boley A, Lodge DJ. Region specific knockdown of Parvalbumin or Somatostatin produces neuronal and behavioral deficits consistent with those observed in schizophrenia. Transl Psychiatry. 2019; 9:264. 10.1038/s41398-019-0603-6 31636253 PMC6803626

[12] Ren Z, Pribiag H, Jefferson SJ, Shorey M, Fuchs T, Stellwagen D, Luscher B. Bidirectional Homeostatic Regulation of a Depression-Related Brain State by Gamma-Aminobutyric Acidergic Deficits and Ketamine Treatment. Biol Psychiatry. 2016; 80:457–68. 10.1016/j.biopsych.2016.02.009 27062563 PMC4983262

[13] Bast T, Pezze M, McGarrity S. Cognitive deficits caused by prefrontal cortical and hippocampal neural disinhibition. Br J Pharmacol. 2017; 174:3211–25. 10.1111/bph.13850 28477384 PMC5595754

[14] Han K, Min J, Lee M, Kang BM, Park T, Hahn J, Yei J, Lee J, Woo J, Lee CJ, Kim SG, Suh M. Neurovascular Coupling under Chronic Stress Is Modified by Altered GABAergic Interneuron Activity. J Neurosci. 2019; 39:10081–95. 10.1523/jneurosci.1357-19.2019 31672788 PMC6978951

[15] Du RH, Tan J, Sun XY, Lu M, Ding JH, Hu G. Fluoxetine Inhibits NLRP3 Inflammasome Activation: Implication in Depression. Int J Neuropsychopharmacol. 2016; 19:pyw037. 10.1093/ijnp/pyw037 27207922 PMC5043644

[16] Hatch R, Young D, Barber V, Griffiths J, Harrison DA, Watkinson P. Anxiety, Depression and Post Traumatic Stress Disorder after critical illness: a UK-wide prospective cohort study. Crit Care. 2018; 22:310. 10.1186/s13054-018-2223-6 30466485 PMC6251214

[17] Nikayin S, Rabiee A, Hashem MD, Huang M, Bienvenu OJ, Turnbull AE, Needham DM. Anxiety symptoms in survivors of critical illness: a systematic review and meta-analysis. Gen Hosp Psychiatry. 2016; 43:23–9. 10.1016/j.genhosppsych.2016.08.005 27796253 PMC5289740

[18] Rabiee A, Nikayin S, Hashem MD, Huang M, Dinglas VD, Bienvenu OJ, Turnbull AE, Needham DM. Depressive Symptoms After Critical Illness: A Systematic Review and Meta-Analysis. Crit Care Med. 2016; 44:1744–53. 10.1097/ccm.0000000000001811 27153046 PMC7418220

[19] Géa LP, Colombo R, da Rosa ED, Antqueviezc B, de Aguiar ÉZ, Hizo GH, Schmidt GB, Oliveira LF, Stein DJ, Rosa AR. Anhedonic-like behavior correlates with IFNγ serum levels in a two-hit model of depression. Behav Brain Res. 2019; 373:112076. 10.1016/j.bbr.2019.112076 31284015

[20] Di Benedetto G, Burgaletto C, Carta AR, Saccone S, Lempereur L, Mulas G, Loreto C, Bernardini R, Cantarella G. Beneficial effects of curtailing immune susceptibility in an Alzheimer’s disease model. J Neuroinflammation. 2019; 16:166. 10.1186/s12974-019-1554-9 31409354 PMC6693231

[21] Flores J, Noël A, Foveau B, Lynham J, Lecrux C, LeBlanc AC. Caspase-1 inhibition alleviates cognitive impairment and neuropathology in an Alzheimer’s disease mouse model. Nat Commun. 2018; 9:3916. 10.1038/s41467-018-06449-x 30254377 PMC6156230

[22] Rangasamy SB, Jana M, Roy A, Corbett GT, Kundu M, Chandra S, Mondal S, Dasarathi S, Mufson EJ, Mishra RK, Luan CH, Bennett DA, Pahan K. Selective disruption of TLR2-MyD88 interaction inhibits inflammation and attenuates Alzheimer’s pathology. J Clin Invest. 2018; 128:4297–312. 10.1172/jci96209 29990310 PMC6159992

[23] Anisman H. Cascading effects of stressors and inflammatory immune system activation: implications for major depressive disorder. J Psychiatry Neurosci. 2009; 34:4–20. 19125209 PMC2612083

[24] Lim L, Mi D, Llorca A, Marín O. Development and Functional Diversification of Cortical Interneurons. Neuron. 2018; 100:294–313. 10.1016/j.neuron.2018.10.009 30359598 PMC6290988

[25] DeFelipe J, López-Cruz PL, Benavides-Piccione R, Bielza C, Larrañaga P, Anderson S, Burkhalter A, Cauli B, Fairén A, Feldmeyer D, Fishell G, Fitzpatrick D, Freund TF, et al. New insights into the classification and nomenclature of cortical GABAergic interneurons. Nat Rev Neurosci. 2013; 14:202–16. 10.1038/nrn3444 23385869 PMC3619199

[26] Mukherjee A, Carvalho F, Eliez S, Caroni P. Long-Lasting Rescue of Network and Cognitive Dysfunction in a Genetic Schizophrenia Model. Cell. 2019; 178:1387–1402.e14. 10.1016/j.cell.2019.07.023 31474363

[27] Gerhard DM, Pothula S, Liu RJ, Wu M, Li XY, Girgenti MJ, Taylor SR, Duman CH, Delpire E, Picciotto M, Wohleb ES, Duman RS. GABA interneurons are the cellular trigger for ketamine’s rapid antidepressant actions. J Clin Invest. 2020; 130:1336–49. 10.1172/jci130808 31743111 PMC7269589

[28] Xu H, Liu L, Tian Y, Wang J, Li J, Zheng J, Zhao H, He M, Xu TL, Duan S, Xu H. A Disinhibitory Microcircuit Mediates Conditioned Social Fear in the Prefrontal Cortex. Neuron. 2019; 102:668–682.e5. 10.1016/j.neuron.2019.02.026 30898376

[29] Murthy S, Kane GA, Katchur NJ, Lara Mejia PS, Obiofuma G, Buschman TJ, McEwen BS, Gould E. Perineuronal Nets, Inhibitory Interneurons, and Anxiety-Related Ventral Hippocampal Neuronal Oscillations Are Altered by Early Life Adversity. Biol Psychiatry. 2019; 85:1011–20. 10.1016/j.biopsych.2019.02.021 31027646 PMC6590696

[30] Wohleb ES, Wu M, Gerhard DM, Taylor SR, Picciotto MR, Alreja M, Duman RS. GABA interneurons mediate the rapid antidepressant-like effects of scopolamine. J Clin Invest. 2016; 126:2482–94. 10.1172/jci85033 27270172 PMC4922686

[31] Kolata SM, Nakao K, Jeevakumar V, Farmer-Alroth EL, Fujita Y, Bartley AF, Jiang SZ, Rompala GR, Sorge RE, Jimenez DV, Martinowich K, Mateo Y, Hashimoto K, et al. Neuropsychiatric Phenotypes Produced by GABA Reduction in Mouse Cortex and Hippocampus. Neuropsychopharmacology. 2018; 43:1445–56. 10.1038/npp.2017.296 29362511 PMC5916365

[32] Czéh B, Varga ZKK, Henningsen K, Kovács GL, Miseta A, Wiborg O. Chronic stress reduces the number of GABAergic interneurons in the adult rat hippocampus, dorsal-ventral and region-specific differences. Hippocampus. 2015; 25:393–405. 10.1002/hipo.22382 25331166

[33] Fuchs T, Jefferson SJ, Hooper A, Yee PH, Maguire J, Luscher B. Disinhibition of somatostatin-positive GABAergic interneurons results in an anxiolytic and antidepressant-like brain state. Mol Psychiatry. 2017; 22:920–30. 10.1038/mp.2016.188 27821870 PMC5422144

[34] Miyata S, Kumagaya R, Kakizaki T, Fujihara K, Wakamatsu K, Yanagawa Y. Loss of Glutamate Decarboxylase 67 in Somatostatin-Expressing Neurons Leads to Anxiety-Like Behavior and Alteration in the Akt/GSK3β Signaling Pathway. Front Behav Neurosci. 2019; 13:131. 10.3389/fnbeh.2019.00131 31275123 PMC6591520

[35] Fee C, Banasr M, Sibille E. Somatostatin-Positive Gamma-Aminobutyric Acid Interneuron Deficits in Depression: Cortical Microcircuit and Therapeutic Perspectives. Biol Psychiatry. 2017; 82:549–559. 10.1016/j.biopsych.2017.05.024 28697889 PMC5610074

[36] Ognjanovski N, Schaeffer S, Wu J, Mofakham S, Maruyama D, Zochowski M, Aton SJ. Parvalbumin-expressing interneurons coordinate hippocampal network dynamics required for memory consolidation. Nat Commun. 2017; 8:15039. 10.1038/ncomms15039 28382952 PMC5384212

[37] Hammer M, Krueger-Burg D, Tuffy LP, Cooper BH, Taschenberger H, Goswami SP, Ehrenreich H, Jonas P, Varoqueaux F, Rhee JS, Brose N. Perturbed Hippocampal Synaptic Inhibition and γ-Oscillations in a Neuroligin-4 Knockout Mouse Model of Autism. Cell Rep. 2015; 13:516–523. 10.1016/j.celrep.2015.09.011 26456829 PMC5862414

[38] Sauer JF, Strüber M, Bartos M. Impaired fast-spiking interneuron function in a genetic mouse model of depression. Elife. 2015; 4:e04979. 10.7554/elife.04979 25735038 PMC4374525

[39] Park K, Lee J, Jang HJ, Richards BA, Kohl MM, Kwag J. Optogenetic activation of parvalbumin and somatostatin interneurons selectively restores theta-nested gamma oscillations and oscillation-induced spike timing-dependent long-term potentiation impaired by amyloid β oligomers. BMC Biol. 2020; 18:7. 10.1186/s12915-019-0732-7 31937327 PMC6961381

[40] Anderson ST, Commins S, Moynagh P, Coogan AN. Chronic fluoxetine treatment attenuates post-septic affective changes in the mouse. Behav Brain Res. 2016; 297:112–5. 10.1016/j.bbr.2015.10.011 26455875

[41] Godavarthi SK, Sharma A, Jana NR. Reversal of reduced parvalbumin neurons in hippocampus and amygdala of Angelman syndrome model mice by chronic treatment of fluoxetine. J Neurochem. 2014; 130:444–54. 10.1111/jnc.12726 24678582

[42] Filipović D, Stanisavljević A, Jasnić N, Bernardi RE, Inta D, Perić I, Gass P. Chronic Treatment with Fluoxetine or Clozapine of Socially Isolated Rats Prevents Subsector-Specific Reduction of Parvalbumin Immunoreactive Cells in the Hippocampus. Neuroscience. 2018; 371:384–94. 10.1016/j.neuroscience.2017.12.020 29275206

[43] Zhang W, Rosenkranz JA. Effects of Repeated Stress on Age-Dependent GABAergic Regulation of the Lateral Nucleus of the Amygdala. Neuropsychopharmacology. 2016; 41:2309–23. 10.1038/npp.2016.33 26924679 PMC4946062

[44] Sagi Y, Medrihan L, George K, Barney M, McCabe KA, Greengard P. Emergence of 5-HT5A signaling in parvalbumin neurons mediates delayed antidepressant action. Mol Psychiatry. 2020; 25:1191–1201. 10.1038/s41380-019-0379-3 30804492 PMC7244406

[45] Méndez P, Pazienti A, Szabó G, Bacci A. Direct alteration of a specific inhibitory circuit of the hippocampus by antidepressants. J Neurosci. 2012; 32:16616–28. 10.1523/jneurosci.1720-12.2012 23175817 PMC6621787

[46] Qiu LL, Pan W, Luo D, Zhang GF, Zhou ZQ, Sun XY, Yang JJ, Ji MH. Dysregulation of BDNF/TrkB signaling mediated by NMDAR/Ca(2+)/calpain might contribute to postoperative cognitive dysfunction in aging mice. J Neuroinflammation. 2020; 17:23. 10.1186/s12974-019-1695-x 31948437 PMC6966800

[47] Ji M, Li S, Zhang L, Gao Y, Zeng Q, Mao M, Yang J. Sepsis induced cognitive impairments by disrupting hippocampal parvalbumin interneuron-mediated inhibitory network via a D4-receptor mechanism. Aging (Albany NY). 2020; 12:2471–84. 10.18632/aging.102755 32019903 PMC7041733

