# Supplementary material for: Parvalbumin interneuron-mediated neural disruption in an animal model of postintensive care syndrome: prevention by fluoxetine
Source: Aging (Albany NY). 2021 Feb 22;13(6):8720–36. doi: 10.18632/aging.202684 (PMC8034944; doi:10.18632/aging.202684)
Supplement: Supplementary Figures [file aging-13-202684-s001.pdf]

SUPPLEMENTARY FIGURES

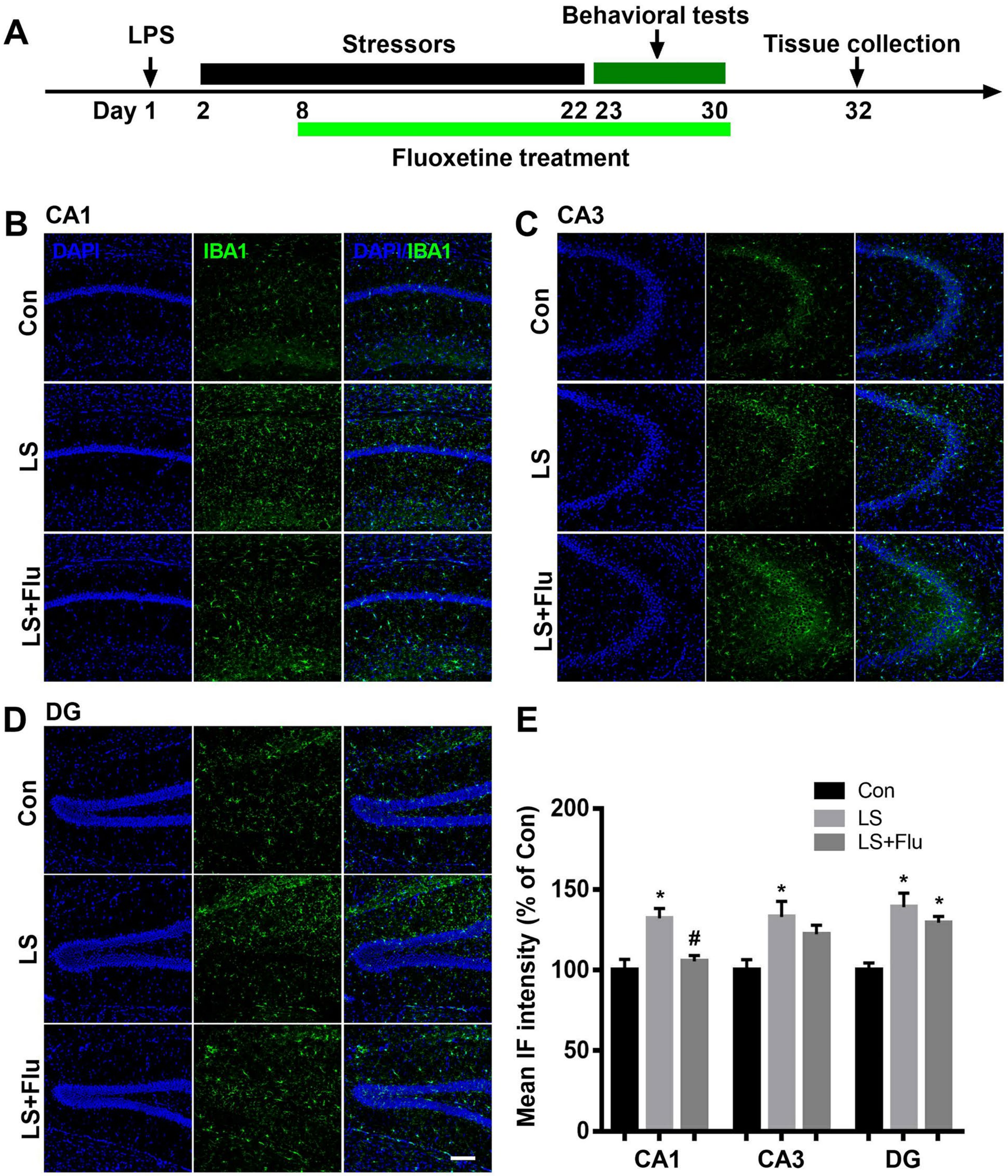

Supplementary Figure 1. Fluoxetine treatment attenuated activation of microglia after combined stress. (**A**) Schematic timeline of the experimental procedure. (**B–D**) Representative images of IBA-1-positive cells in all subregions of the hippocampus. (**E**) Quantification of mean IBA-1 immunofluorescence in the hippocampus. Data are shown as mean ± SEM (n = 4), $^{*}P < 0.05$ vs control group, $^{\#}P < 0.05$ vs LS group, scale bar = 100 μm. Con, control; LPS, lipopolysaccharide; Flu, fluoxetine; IF, immunofluorescence; LS, LPS + stress.

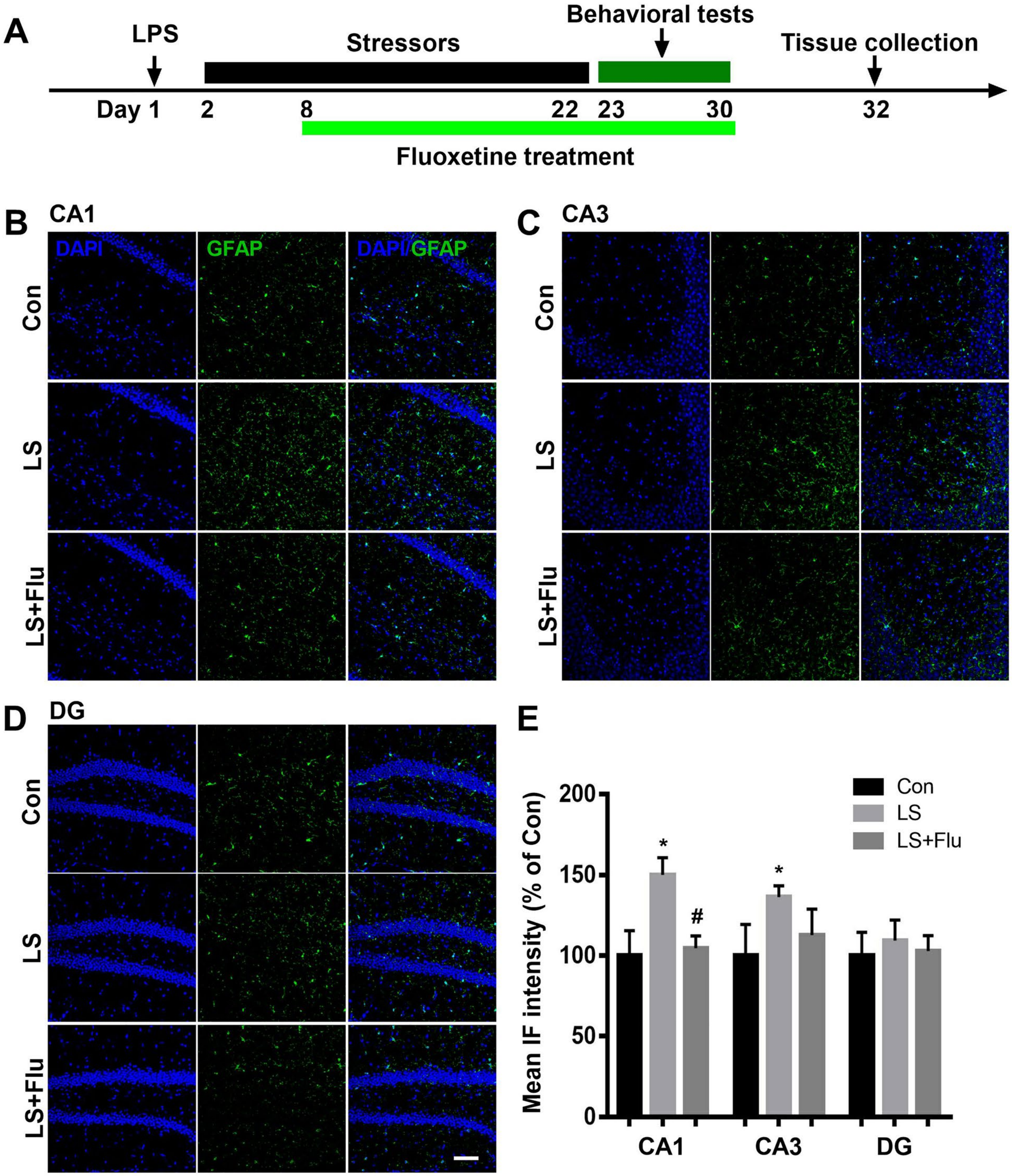

Supplementary Figure 2. Fluoxetine treatment attenuated activation of astrocytes after combined stress. (**A**) Schematic timeline of the experimental procedure. (**B–D**) Representative images of GFAP-positive cells in all subregions of the hippocampus. (**E**) Quantification of mean GFAP immunofluorescence in the hippocampus. Data are shown as mean ± SEM (n = 4), $^{*}P < 0.05$ vs control group, $^{\#}P < 0.05$ vs LS group, scale bar = 100 μm. Con, control; LPS, lipopolysaccharide; Flu, fluoxetine; IF, immunofluorescence; LS, LPS + stress.
