# Supplementary material for: Parvalbumin interneuron-mediated neural disruption in an animal model of postintensive care syndrome: prevention by fluoxetine
Source: Aging (Albany NY). 2021 Feb 22;13(6):8720–36. doi: 10.18632/aging.202684 (PMC8034944; doi:10.18632/aging.202684)
Supplement: Supplementary Table 1 [file aging-13-202684-s002.pdf]

SUPPLEMENTARY TABLES

Supplementary Table 1. Chronic stress protocol.

	Day 1	Day 2	Day 3	Day 4	Day 5	Day 6	Day 7
**Week 1**	C, D, G, H	A, C, F, G	B, D, F, H	A, B, C, G	B, D, E, F	A, D, F, H	B, C, F, G
**Week 2**	A, B, E, H	A, C, E, G	B, C, D, G	A, B, D, H	C, E, F, G	A, E, G, H	A, E, F, G
**Week 3**	B, C, F, G	B, E, G, H	A, D, F, G	A, C, G, H	A, B, D, E	B,C, D, H	C, D, F, H

(**A**) light on overnight (12 h). (**B**) physical restraint for 6 h. (**C**) cage tilt 45°C for 12 h. (**D**) lights-off for 3 h during the daylight phase, (**E**) wet bedding overnight, (**F**) odor overnight, (**G**) noise in the room for 12 h, (**H**) food and water deprivation overnight.
